# Phase Difference Measurement of Under-Sampled Sinusoidal Signals for InSAR System Phase Error Calibration

**DOI:** 10.3390/s19235328

**Published:** 2019-12-03

**Authors:** Zhihui Yuan, Yice Gu, Xuemin Xing, Lifu Chen

**Affiliations:** 1 Laboratory of Radar Remote Sensing Applications, Changsha University of Science and Technology, Changsha 410114, China; yuanzhihui@csust.edu.cn (Z.Y.); guyice@stu.csust.edu.cn (Y.G.); lifu_chen@csust.edu.cn (L.C.); 2 School of Electrical and Information Engineering, Changsha University of Science and Technology, Changsha 410114, China; 3 Roy M. Huffington Department of Earth Sciences, Southern Methodist University, Dallas, TX 75275, USA; 4 School of Traffic and Transportation Engineering, Changsha University of Science and Technology, Changsha 410114, China

**Keywords:** interferometric synthetic aperture radar (InSAR), phase error calibration, phase difference measurement, under-sampling, coherent accumulation

## Abstract

Phase difference measurement of sinusoidal signals can be used for phase error calibration of the spaceborne single-pass interferometric synthetic aperture radar (InSAR) system. However, there are currently very few papers devoted to the discussion of phase difference measurement of high-frequency internal calibration signals of the InSAR system, especially the discussion of sampling frequency selection and the corresponding measuring method when the high-frequency signals are sampled under the under-sampling condition. To solve this problem, a phase difference measurement method for high-frequency sinusoidal signals is proposed, and the corresponding sampling frequency selection criteria under the under-sampling condition is determined. First, according to the selection criteria, the appropriate under-sampling frequency was chosen to sample the two sinusoidal signals with the same frequency. Then, the sampled signals were filtered by limited recursive average filtering (LRAF) and coherently accumulated in the cycle of the baseband signal. Third, the filtered and accumulated signals were used to calculate the phase difference of the two sinusoidal signals using the discrete Fourier transform (DFT), digital correlation (DC), and Hilbert transform (HT)-based methods. Lastly, the measurement accuracy of the three methods were compared respectively by different simulation experiments. Theoretical analysis and experiments verified the effectiveness of the proposed method for the phase error calibration of the InSAR system.

## 1. Introduction

Phase difference measurement of sinusoidal signals [1,2,3,4,5,6,7,8,9] is one of the most important research topics in applications such as phase error calibration of the spaceborne single-pass interferometric synthetic aperture radar (InSAR) system [10,11,12,13], power system monitoring [14], radio frequency communication [15], and laser ranging [16]. For the spaceborne single-pass InSAR system, a possible interferometric phase error can arise from relative phase differences between the two receiver channels, because the two signal receivers are not identical mechanically or thermally, and the signal path length from receiving antenna to electronics is vastly different because of the 60 m baseline [12]. Therefore, an internal calibration signal with common reference is distributed to the antennas over an optical fiber cable to the deployed antenna [10,11,12,13], and the phase difference of the internal calibration signals (usually sinusoidal signals) received separately from the primary and secondary antennas needs to be measured. More than that, the frequency of the calibration signal is generally high. For example, the frequency of the calibration signal of the InSAR system on the Shuttle Radar Topography Mission (SRTM) is as high as 263 MHz [10]. Due to the limitation of the A/D converter itself, the sampling frequency cannot be made too high, so the signal can only be sampled by under-sampling [17].

Regarding the phase difference measurement of sinusoidal signals, many different methods have been proposed, including discrete Fourier transform (DFT) [18,19], digital correlation (DC) [20], Hilbert transform (HT) [21], least squares (LS) [22], independent component analysis (ICA) [23], and zero cross detection (ZCD) [24] based methods. In Reference [18], considering the negative frequency contribution, a new DFT-based algorithm for phase difference measurement of extreme frequency signal is proposed. The phase difference calculation formula under different windows is deduced in detail. Compared with the traditional DFT-based phase difference measurement algorithm, the new algorithm has stronger spectral leakage suppression capability and higher precision. In Reference [19], considering the spectral superposition of real signals, a new modulation and DFT-based estimation method is proposed which obtains the phase difference by combining the estimated signal frequency and four DFT samples of the modulated signal. However, the above DFT-based phase difference measurement methods have a drawback in that a complete sampling cycle is required for calculation. In Reference [20], an all-digital phase measurement method based on cross-correlation analysis is proposed, and the measurement errors caused by sampling quantization, intrinsic white noise, and non-whole-cycle sampling are analyzed. This method is named the digital correlation (DC)-based method in this paper. In Reference [21], a phase difference estimation method based on data expansion and HT is proposed. This method obtains the phase difference estimation by data expansion, HT, cross-correlation, autocorrelation, and weighted phase averaging which can suppress the end effect of the HT effectively. In Reference [22], a new algorithm for phase difference measurement of sinusoidal signals based on LS is proposed. The algorithm uses digitized samples of the input signal and can determine the amplitude and phase of the two signals simultaneously. Compared with the DFT-based method, this algorithm not only has the advantages of good filtering characteristics and high precision, but also filters out high-frequency components, direct current components, and white noise and can adjust the length of the data window according to the requirements of accuracy and calculation speed. In Reference [23], a robust phase difference measurement method is proposed which uses ICA to separate sinusoidal signals and noise and has strong robustness and accuracy. The ZCD-based method proposed in Reference [24] has a relatively simple principle and is relatively easy to implement in hardware and software, but it is susceptible to interference from noise and harmonics and has poor real-time performance.

However, there are currently very few papers devoted to the discussion of phase difference measurement of high-frequency internal calibration signals of the InSAR system, especially the discussion of sampling frequency selection when the high-frequency signals are sampled under the under-sampling condition. Under such conditions, the initial phases of the sampled signal and the original high-frequency internal calibration signal will be the same, opposite or irrelevant which is different from the general situation. Therefore, the selection of the sampling frequency becomes very important.

In response to the problems mentioned above, the phase difference measurement of high-frequency sinusoidal signals is discussed in this paper, and the corresponding sampling frequency selection criteria under the under-sampling condition is also determined. According to the previous analysis, the DFT-based method is the classical frequency domain measurement method which can be realized by fast Fourier transform (FFT) and can effectively suppress the influence of random noise and harmonics. The DC-based method is the classical time domain measurement method which has a strong ability to suppress random noise; the HT-based method can make real-time measurement of phase difference, and, with the progress of the computer and signal processing technology, the method will continue to overcome the difficulty in instrument design and improve the measurement accuracy. In view of the advantages and representativeness of these three methods, we chose to apply them to the phase difference measurement of high-frequency signals in the phase error calibration of the InSAR system and analyzed and compared them. The specific application process was as follows: Firstly, according to the selection criteria, the appropriate under-sampling frequency was chosen to sample the two sinusoidal signals with the same frequency. Then, the sampled signals were filtered by the limited recursive average filtering (LRAF) and coherently accumulated in the cycle of the baseband signal. Thirdly, the filtered and accumulated sampled signals were used to calculate the phase difference of the two sinusoidal signals by using the DFT-, DC-, and HT-based methods. Lastly, the measurement accuracy of the three methods were compared, respectively, by the different simulation experiments. The experimental results showed that the proposed method in this paper is suitable for the phase difference measurement of the high-frequency internal calibration signals in the InSAR system and can improve the accuracy of the phase difference measurement results.

## 2. Selection of Sampling Frequency

In this section, the selection criteria of the sampling frequency for the sinusoidal signal under the under-sampling condition is deduced by mathematical formulas and diagrams.

Considering a sinusoidal signal s(t) and its mathematical expression:

(1) s(t)=Acos(2πft+φ)

where A is the unknown amplitude, f the frequency, t the time, and φ the unknown initial phase (−π<φ≤π). Assuming that the sinusoidal signal is sampled with the frequency $f_{s}$, it can be known from the Nyquist sampling theorem that $f_{s}$ must be greater than or equal to 2f to accurately recover the original signal. Especially when it is necessary to measure the phase difference between two sinusoidal signals, $f_{s}$ must be much larger than 2f. However, when the signal frequency itself is very high, as the signal frequency increases, the sampling frequency will also become higher and higher. When the sampling frequency is high to a certain extent, it will be difficult to achieve under the existing equipment and technical conditions, which makes it difficult to sample the high frequency signal. Therefore, it is necessary to reduce the sampling frequency according to the band-pass sampling theorem [25], that is, to use the under-sampling method to sample the signal. Next, we will discuss the selection of the sampling frequency and its value range.

**Figure 1:**
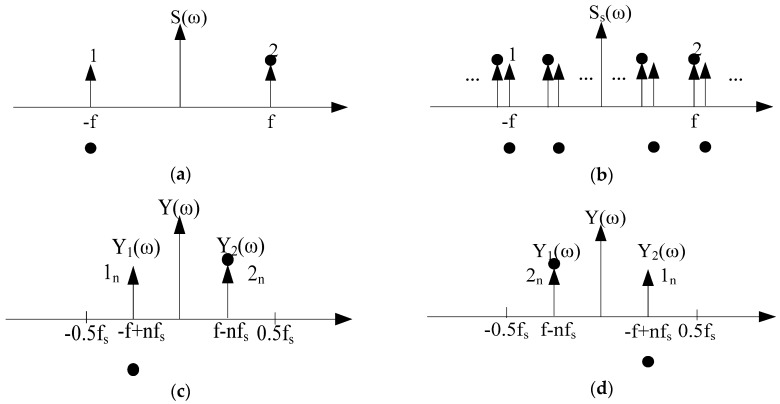
Signal spectrum schematic: (**a**) Original signal spectrum; (**b**) signal spectrum after sampling; (**c**) signal spectrum after low-pass filtering (case 1); (**d**) signal spectrum after low-pass filtering (case 2).

The spectrum of the signal s(t) is shown in Figure 1a, where ω means the angular frequency, f is the frequency of the signal, the vertical upward arrow represents the amplitude spectrum, and the solid black dot represents the phase spectrum. Figure 1b is the spectrum of the sampled signal $s_{s}\left(t\right)$. The spectral expression of the sampled signal, $s_{s}\left(t\right)$, is as follows:

(2) $$\begin{array}{ll} S_{s}\left(\omega \right) & =\left(\pi e^{-j\phi }\delta \left(\omega +2\pi f\right)+\pi e^{j\phi }\delta \left(\omega -2\pi f\right)\right)*\\ & f_{s}\sum_{n=-\infty }^{+\infty }\delta \left(\omega -n\cdot 2\pi f_{s}\right)\\ & =\pi f_{s}e^{-j\phi }\sum_{n=-\infty }^{+\infty }\delta \left(\omega +2\pi f-n\cdot 2\pi f_{s}\right)+\\ & \pi f_{s}e^{j\phi }\sum_{n=-\infty }^{+\infty }\delta \left(\omega -2\pi f-n\cdot 2\pi f_{s}\right) \end{array}$$

Obviously, in order to avoid spectral aliasing of the sampled signal, the following condition must be met between the sampling frequency, $f_{s}$, and the signal frequency, f:

(3) $$-f+nf_{s}\neq f,n=1,2,3,\cdots$$

That is:

(4) $$f_{s}\neq \frac{2f}{n}=\frac{f}{n/2},n=1,2,3,\cdots$$

After passing through a filter with a gain of $1/f_{s}$ and a passband range of $0∼0.5f_{s}$, the rest is the spectrum of the baseband signal. At this time, there may be two cases, as shown in Figure 1c,d, where the part marked with “1*_n_*” is the result of shifting the spectrum of the original signal to the right by *n* times, and the part marked with “2*_n_*” is the result of shifting the spectrum of the original signal by *n* times.

(1) In the case shown in Figure 1c, the condition as follows must be met:

(5) $$0<f-nf_{s}<0.5f_{s},n=1,2,3,\cdots$$

That is:

(6) $$\frac{f}{n+0.5}<f_{s}<\frac{f}{n},n=1,2,3,\cdots$$

The resulting baseband signal spectrum at this time is:

(7) $$Y\left(\omega \right)=\pi e^{-j\phi }\delta \left(\omega +2\pi f-2\pi nf_{s}\right)+\pi e^{j\phi }\delta \left(\omega -2\pi f+2\pi nf_{s}\right)$$

The reconstructed baseband signal after inverse Fourier transform is:

(8) $$y\left(t\right)=\cos\left(2\pi \left(f-nf_{s}\right)t+\varphi \right)=\cos\left(2\pi f_{0}t+\varphi_{0}\right)$$

where $f_{0}$ is the frequency of y(t) and $\varphi_{0}$ is the initial phase of y(t). Then, as can be seen from Equation (8):

(9) $$\left\{ \begin{array}{l} f_{0}=f-nf_{s}\\ \varphi_{0}=\varphi \end{array}\right.$$

That is to say, the initial phase of the baseband signal, y(t), is the same as the initial phase of the signal s(t).

(2) In the case shown in Figure 1d, the condition as follows must be met:

(10) $$0<-f+nf_{s}<0.5f_{s},n=1,2,3,\cdots$$

That is:

(11) $$\frac{f}{n}<f_{s}<\frac{f}{n-0.5},n=1,2,3,\cdots$$

The resulting baseband signal spectrum at this time is:

(12) $$Y\left(\omega \right)=\pi e^{-j\phi }\delta \left(\omega -2\pi f+2\pi nf_{s}\right)+\pi e^{j\phi }\delta \left(\omega +2\pi f-2\pi nf_{s}\right)$$

The reconstructed baseband signal after inverse Fourier transform is:

(13) $$y\left(t\right)=\cos\left(-2\pi \left(f-nf_{s}\right)t-\varphi \right)=\cos\left(2\pi f_{0}t+\varphi \right)$$

As can be seen from Equation (13):

(14) $$\left\{ \begin{array}{l} f_{0}=-f+nf_{s}\\ \varphi_{0}=-\varphi \end{array}\right.$$

That is to say, the initial phase of the baseband signal, y(t), is opposite to the initial phase of the signal, s(t).

From the above analysis, the following conclusions can be drawn: high-frequency sinusoidal signals can be reconstructed based on the frequency and initial phase of the low frequency baseband signal, and the phase difference of the two sinusoidal signals with the same frequency can be measured by selecting the sampling frequency that satisfies the conditions of Equations (6) or (11).

## 3. Signal Processing Based on Limited Recursive Average Filtering and Coherent Accumulation

In this section, the signal processing process based on limited recursive average filtering (LRAF) and coherent accumulation (CA) under under-sampling conditions is discussed. For a detection system, the preprocessing of the collected signals is an essential part in the whole measurement process. If we want to measure the phase difference, the collected signals should be preprocessed to eliminate the effects of the noise to some extent. In order to minimize the influence of the noise on the phase difference measurement, the preprocessing step used in this paper is divided into two parts: LRAF and CA.

### 3.1. Signal Sampling

For the case where the frequency of the calibration signal in the InSAR system is high, under-sampling should be selected to sample the signal according to the band-pass sampling theorem [25]. Therefore, the two sinusoidal signals with the same frequency can be sampled by selecting the appropriate sampling frequency according to the selection criteria described in Section 2. Here, we assume that the sampling frequency satisfies the condition in Equation (6), the total length of the sampled signal is N points, the number of sampling points in the baseband signal’s period is $N_{0}$, and the relationship between N and $N_{0}$ is $N=m\cdot N_{0}$ (m is a positive integer). Then, the two sampled signals are:

(15) $$\begin{array}{ll} {\hat{s}}_{1}\left(kT\right) & =A_{1}\cos\left(2\pi \left(f_{0}+nf_{s}\right)kT+\varphi_{1}\right)+n_{1}(kT)\\ & =A_{1}\cos\left(2\pi f_{0}kT+2\pi nk+\varphi_{1}\right)+n_{1}(kT)\\ & =A_{1}\cos\left(2\pi f_{0}kT+\varphi_{1}\right)+n_{1}(kT),\;k=0,\;1,\;2,\cdots ,\;N \end{array}$$

(16) $$\begin{array}{ll} {\hat{s}}_{2}\left(kT\right) & =A_{2}\cos\left(2\pi \left(f_{0}+nf_{s}\right)kT+\varphi_{2}\right)++n_{2}(kT)\\ & =A_{2}\cos\left(2\pi f_{0}kT+2\pi nk+\varphi_{2}\right)+n_{2}(kT)\\ & =A_{2}\cos\left(2\pi f_{0}kT+\varphi_{2}\right)+n_{2}(kT),\;k=0,\;1,\;2,\cdots ,\;N \end{array}$$

where *T* is the sampling period ($T=1/f_{s}$), $n_{1}(kT)$ and $n_{2}(kT)$ are the noises of the two receiving channels, and the physical meaning of other parameters are shown in the explanation part of Equation (1) in Section 1.

### 3.2. Limited Recursive Average Filtering

There are many ways to remove signal noise, including the seasonal model method, autoregressive summation moving average model method, limited recursive average filtering method, etc. In this paper, the LRAF method was used to deal with high-frequency interference. In this method, $N_{w}$ sampling points continuously obtained from each receiving channel were treated as a queue; then, the abnormal sampling points with clearly distorted amplitudes were deleted according to the preset threshold, and then the remaining sampling points in the queue were arithmetically averaged. The calculated arithmetic average value was taken as the new sample value of the sampling point at the center of the queue, so that the filtering function was implemented. The process was done point by point. When a new sampling point was obtained, it was placed at the end of the queue, and the sampling point at the beginning of the original queue (first in first out, FIFO) was discarded, and then the same operation as before was performed.

The specific steps for performing the LRAF process on $s_{1}\left(kT\right)$ and $s_{2}\left(kT\right)$ are as follows:

(1) Observing the characteristics of the sampling signals from the two receiving channels, determining the maximum allowable amplitude difference among adjacent sampling points, respectively, recorded as the threshold values $A_{th1}$ and $A_{th2}$;
(2) The length a of the queue, $N_{w}$, is determined based on the total number of samples in a baseband signal period;
(3) From the first sampling point, the limited average filtering is performed point by point. The queue corresponding to the *i*th sampling point is $\left[i-N_{w}/2,\;\cdots ,\;i,\;\cdots ,\;i+N_{w}/2\right]$, the abnormal sampling points whose amplitudes are clearly distorted are deleted according to $A_{th1}$ and $A_{th2}$, then the remaining sampling points in the queue are arithmetically averaged, and then the calculated arithmetic average value is taken as the new sample value of the *i*th sampling point.

### 3.3. Coherent Accumulation

Coherent accumulation refers to the addition or accumulation of the signal-to-noise ratio equal to the signal-to-noise ratio of a single pulse multiplied by the pulse number of the pulse train. In this paper, a pulse was equivalent to a signal with a baseband period length. Theoretically, CA improves the signal-to-noise ratio by a factor of *N* (*N* is the number of accumulated pulses). By coherently accumulating the filtered signal with the period $T_{0}\left(T_{0}=N_{0}/f_{s}\right)$ of the baseband signal, y(t), more Gaussian noise can be further filtered out, i.e.,:

(17) $$\begin{array}{cc} {\hat{s}}_{1a}\left(kT\right) & =A_{1}\cos\left(2\pi f_{0}kT+\varphi_{1}\right)+A_{1}\cos\left(2\pi f_{0}\left(k+N_{0}\right)T+\varphi_{1}\right)\\ & +\cdots +A_{1}\cos\left(2\pi f_{0}\left(k+\left(m-1\right)N_{0}\right)T+\varphi_{1}\right)+n_{1a}(kT),\\ k & =0,\;1,\;2,\cdots ,\;N_{0}-1 \end{array}$$

(18) $$\begin{array}{cc} {\hat{s}}_{2a}\left(kT\right) & =A_{2}\cos\left(2\pi f_{0}kT+\varphi_{2}\right)+A_{2}\cos\left(2\pi f_{0}\left(k+N_{0}\right)T+\varphi_{2}\right)\\ & +\cdots +A_{2}\cos\left(2\pi f_{0}\left(k+\left(m-1\right)N_{0}\right)T+\varphi_{2}\right)+n_{2a}(kT),\\ k & =0,\;1,\;2,\cdots ,\;N_{0}-1 \end{array}$$

Most of the noise interference was already filtered out at this time, so the filtered signals, ${\hat{s}}_{1a}\left(kT\right)$ and ${\hat{s}}_{2a}\left(kT\right)$, can be directly used for the next processing step: phase difference measurement.

## 4. Phase Difference Measurement

At present, the measurement methods used to estimate the phase difference between two sinusoidal signals can be divided into two categories. The first category is the model-based parametric measurement algorithm, such as the LS, HT, and correlation analysis methods. The second is the model-based non-parametric measurement algorithm, such as the DFT method. In this paper, the DFT, DC, and HT methods were used to measure the phase difference of the signals that were processed by LRAF and CA, and the performance of these methods are compared and analyzed in Section 5. Below we introduce the three methods separately.

### 4.1. DFT-Based Method

Among the many phase difference measurement methods, the DFT-based method is widely used because of its physical meaning, simple implementation, high measurement accuracy, and fast response speed. This method can transform the signal from the time space to frequency domain and can effectively suppress the influence of random noise and harmonics. The DFT operations are performed on the accumulated signals $s_{1a}\left(kT\right)$ and $s_{2a}\left(kT\right)$ separately, so that the initial phases $\varphi_{1}$ and $\varphi_{2}$ of the two sinusoidal signals can be obtained by:

(19) $$\begin{array}{ll} \varphi_{1} & =∠\left\{{\mathrm{DFT}\left({\hat{s}}_{1a}\left(nT\right)\right)|}_{k=1}\right\}\\ & =∠\left\{{\left(\sum_{n=0}^{N_{0}-1}{\hat{s}}_{1a}\left(nT\right)e^{-j\frac{2\pi }{N_{0}}nk}\right)|}_{k=1}\right\} \end{array}$$

(20) $$\begin{array}{ll} \varphi_{2} & =∠\left\{{\mathrm{DFT}\left({\hat{s}}_{2a}\left(nT\right)\right)|}_{k=1}\right\}\\ & =∠\left\{{\left(\sum_{n=0}^{N_{0}-1}{\hat{s}}_{2a}\left(nT\right)e^{-j\frac{2\pi }{N_{0}}nk}\right)|}_{k=1}\right\} \end{array}$$

Then, the phase difference between the two sinusoidal signals is obtained based on the initial phase of the two sinusoidal signals:

(21) $$\varphi =\varphi_{2}-\varphi_{1}$$

### 4.2. DC-Based Method

The DC is a digitized version of the correlation analysis method. In the DC-based method, because the correlation between the noise signal and the effective signal is very small, the method has a good noise suppression ability. Using correlation analysis to calculate the phase difference is considered to be one of the optimal phase difference calculation methods which has the advantages of fast calculation speed, strong anti-noise interference ability, and high accuracy. In this method, the phase difference is obtained by sampling the two noised sinusoidal signals in a full cycle and then performing cross-correlation operations on them. The analytical expression for the cross-correlation operation of the two signals is as follows:

(22) $$R_{xy}\left(\tau \right)=\frac{1}{T_{0}}\int_{0}^{T_{0}}{\hat{s}}_{1a}\left(t\right){\hat{s}}_{2a}\left(t+\tau \right)dt$$

where $R_{xy}\left(\tau \right)$ is the correlation coefficient of the two signals ${\hat{s}}_{1a}\left(t\right)$ and ${\hat{s}}_{2a}\left(t\right)$, τ is the time delay between the two signals, $T_{0}$ is the period of the baseband signal y(t). Ideally, the signal and noise are not related to each other, and the noises of the two receiving channels are also uncorrelated. Therefore, when τ=0, the correlation coefficient $R_{xy}\left(\tau \right)$ will reach the maximum value, and its expression can be simplified as:

(23) $$R_{xy}\left(0\right)=\frac{A_{1}A_{2}}{2}\cos\left(\varphi_{2}-\varphi_{1}\right)$$

Thus, the phase difference between the two sinusoidal signals is:

(24) $$\varphi =\varphi_{2}-\varphi_{1}=\arccos\left(\frac{2R_{xy}\left(0\right)}{A_{1}A_{2}}\right)$$

### 4.3. HT-Based Method

The HT-based method can make real-time measurement of the phase difference and improve the measurement accuracy. The HT technology was successfully applied to the instantaneous frequency measurement of signals very early, but its application to phase difference measurement is rarely seen. The phase difference measurement method based on HT can make real-time measurements of phase difference, and with the progress of computer and signal processing technology, the method will continue to overcome the difficulty in instrument design and improve the measurement accuracy. Therefore, it is more suitable for intelligent detection equipment and other modern detection equipment.

Suppose that the HT of $s_{1a}\left(kT\right)$ and $s_{2a}\left(kT\right)$ are $y_{1}\left(t\right)$ and $y_{2}\left(t\right)$, respectively, and let:

(25) $$z_{1}\left(t\right)=s_{1a}\left(kT\right)\times y_{2}\left(t\right)$$

(26) $$z_{2}\left(t\right)=s_{2a}\left(kT\right)\times y_{1}\left(t\right)$$

(27) $$z=z_{1}\left(t\right)-z_{2}\left(t\right)$$

(28) $$r_{1}\left(t\right)=s_{1a}\left(kT\right)\times s_{2a}\left(kT\right)$$

(29) $$r_{2}\left(t\right)=y_{1}\left(t\right)\times y_{2}\left(t\right)$$

(30) $$r=r_{1}\left(t\right)+r_{2}\left(t\right)$$

At last, the phase difference between the two sinusoidal signals can be obtained by:

(31) $$\varphi =\varphi_{2}-\varphi_{1}=\mathrm{arc}tg\frac{z}{r}$$

## 5. Experiments and Results

In order to verify the effectiveness of the method proposed in this paper, some experiments were carried out using simulated data. The parameters used in the experiments are shown in Table 1.

**Figure 2:**
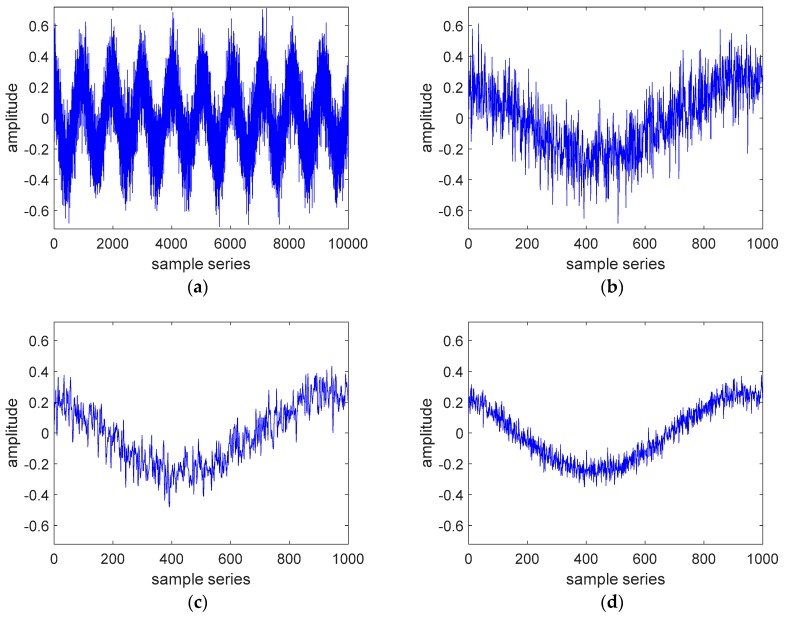
Comparison of the signals before and after limited recursive average filtering and coherent accumulation: (**a**) simulated sinusoidal signal with noise; (**b**) zoomed-in view of one cycle of (**a**); (**c**) one cycle of the filtered signal by limited recursive average filtering (LRAF); (**d**) one cycle of the filtered signal by coherent accumulation (CA).

One of the two simulated sinusoidal signals with noise is shown in Figure 2a. Figure 2b shows the zoomed-in view of one cycle of Figure 2a. Figure 2c is one cycle of the signal filtered by LRAF, and Figure 2d is one cycle of the signal filtered by CA. Comparing Figure 2c,d with Figure 2b, respectively, it can be seen that both the LRAF and CA have obvious filtering effects, because the noise is greatly weakened, but the effect of CA is better than the LRAF.

**Figure 3:**
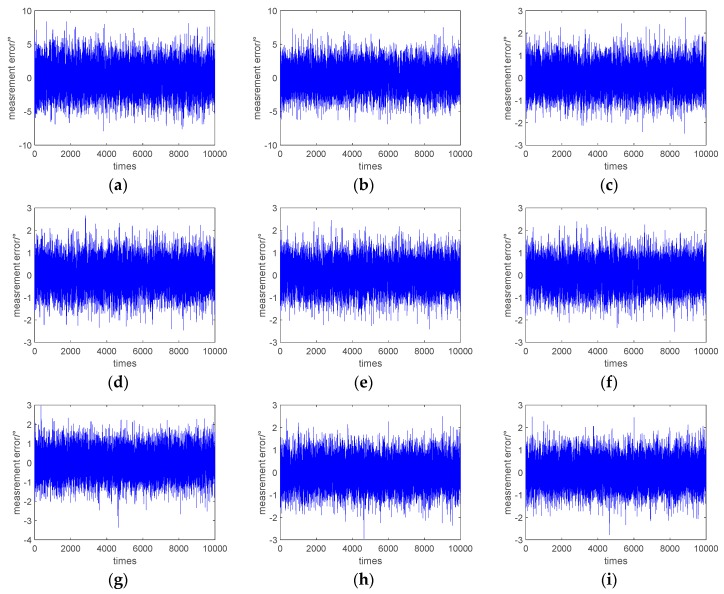
Phase difference measurement error by discrete Fourier transform (DFT)-, digital correlation (DC)-, Hilbert transform (HT) based methods before and after the limited recursive average filtering and coherent accumulation: (**a**) measurement error of the traditional DFT method; (**b**) measurement error of the DFT method after performing the limited recursive average filtering; (**c**) measurement error of the DFT method after performing coherent accumulation; (**d**) measurement error of the traditional DC method; (**e**) measurement error of the DC method after performing the limited recursive average filtering; (**f**) measurement error of the DC method after performing coherent accumulation; (**g**) measurement error of the HT method; (**h**) measurement error of the HT method after performing the limited recursive average filtering; (**i**) measurement error of the HT method after performing the coherent accumulation.

Ten thousand phase difference measurement simulation experiments were carried out, and the phase difference measurement errors by the DFT, DC, and HT-based methods before and after the LRAF and CA are shown in Figure 3. Figure 3a shows the measurement error of the conventional DFT-based method, Figure 3b shows the measurement error of the DFT-based method after performing the LRAF, and Figure 3c shows the measurement error of the DFT-based method after performing the CA. Figure 3d shows the measurement error of the DC-based method, Figure 3e shows the measurement error of the DC-based method after performing the LRAF, and Figure 3f shows the measurement error of the DC-based method after performing the CA. Figure 3g shows the measurement error of the HT-based method, Figure 3h shows the measurement error of the HT-based method after performing the LRAF, and Figure 3i shows the measurement error of the HT-based method after performing the CA. It can be seen from Figure 3a–c that the preprocessing of the received signal had the most obvious effect on the DFT-based method for the measurement accuracy improvement, and the coherent accumulation had a significant effect which reduced the error by five times, but the LRAF had no effect at all. However, the contribution of these two filtering strategies to the DC- and HT-based methods was not as obvious as the DFT-based method. From Figure 3d–i, we know that the phase difference measurement accuracy of the DC- and HT-based methods had only a certain degree of improvement after the LRAF and CA completed, and the degree of improvement for the two methods was similar.

**Figure 4:**
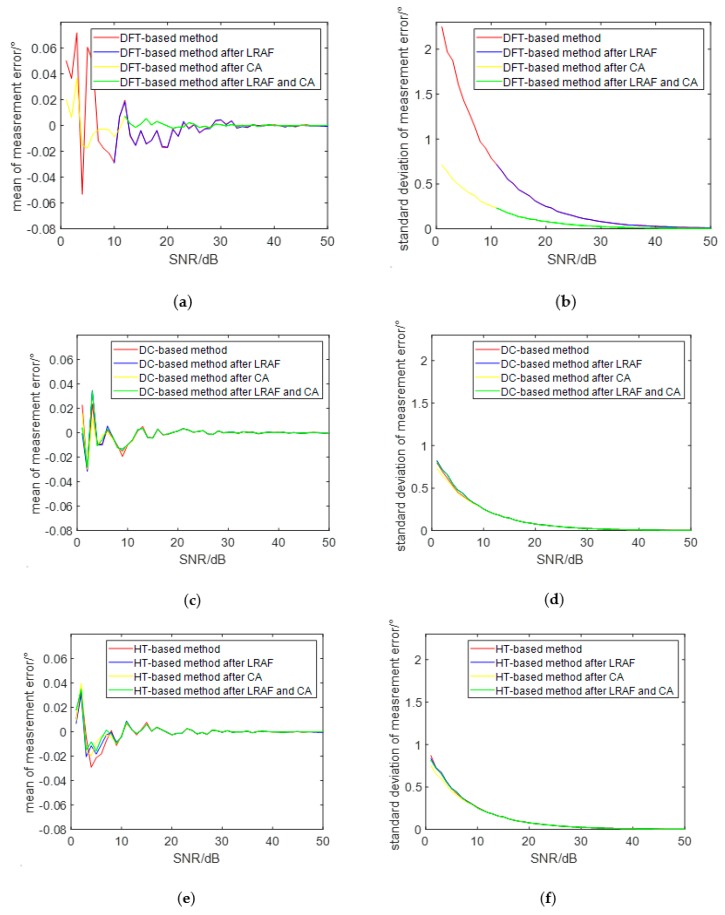
The effect of the preprocessing on the performance of the DFT-, DC-, and HT-based phase difference measurement methods with different SNR: (**a**) mean of the measurement error of the DFT-based method; (**b**) standard deviation of the measurement error of the DFT-based method; (**c**) mean of the measurement error of the DC-based method; (**d**) standard deviation of the measurement error of the DC-based method; (**e**) mean of the measurement error of the HT-based method; (**f**) standard deviation of the measurement error of the HT-based method.

Figure 4 shows the effect of the preprocessing on the performance of the DFT-, DC-, and HT-based phase difference measurement methods under different SNRs. In this experiment, the total number of accumulation cycles was 10, and the SNR varied from 1 dB to 50 dB. Figure 4a,b shows the mean and standard deviation of the measurement error of the phase difference which is measured by the DFT-based method after adding different preprocessing steps, respectively. It can be seen from the two figures that, when the SNR varies from 1 dB to 50 dB, the mean and standard deviation of the measurement error gradually decreased and approached zero at last. However, the measurement accuracy was not improved after the two received signals were filtered by the LRAF, but it was greatly improved after the two received signals were filtered by the CA. More than that, the measurement error of the phase difference was almost negligible when the SNR was greater than 12 dB. Therefore, we can conclude that the CA is very helpful for the performance improvement of the DFT-based phase difference measurement method if the SNR of the signal is poor, while LRAF does not make much sense. Figure 4c,d shows the mean and standard deviation of the measurement error of the phase difference which is measured by the DC-based method after adding different preprocessing steps, respectively. Figure 4e,f shows the mean and standard deviation of the measurement error of the phase difference which is measured by the HT-based method after adding different preprocessing steps, respectively. From Figure 4c–f, we know that the phase difference measurement accuracy of the DC- and HT-based methods is better than the DFT-based method, but it has only a certain degree of improvement after the LRAF and CA are completed, and the degree of improvement for the two methods is similar. Similar to the DFT-based method, the measurement error of the phase difference is almost negligible when the SNR is greater than 12 dB. Therefore, we can conclude that LRAF and CA do not contribute much to the performance improvement of the CA- and HT-based phase difference measurement methods. In general, when the signal-to-noise ratio of the signal is greater than 12 dB, the phase difference measurement can be directly performed using the DFT-, DC-, and HT-based methods.

**Figure 5:**
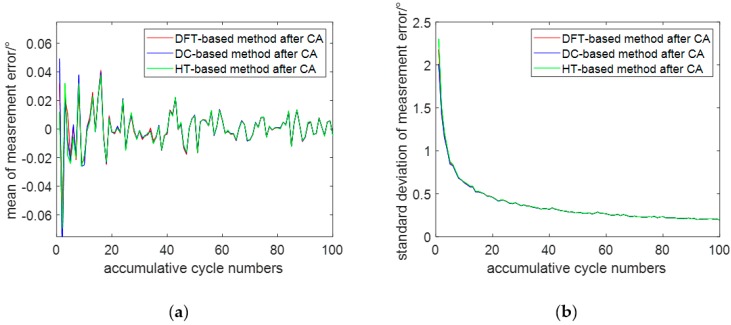
Effects of the different accumulation cycles on each method: (**a**) mean of measurement error; (**b**) standard deviation of measurement error.

Figure 5a,b show the mean and standard deviation of the phase difference measurement error with a SNR of 2 dB and an accumulative cycle number from 1 to 100, respectively. As can be seen from Figure 5a,b, the mean and standard deviation of the phase error also become smaller and smaller as the accumulative cycle number increases, and even negligible when the accumulative cycle number is greater than 20.

Table 2 shows the mean and standard deviation of the measurement error by different phase difference measurement methods with a SNR of 2 dB and an accumulative cycle number of 10. As can be seen from the table, the measurement accuracy was improved after LRAF and CA compared with the direct measurement of the phase difference of the original sinusoidal signal. However, it can also be seen that LRAF had no effect on the DFT-based method but had an effect on the other two phase difference measurement methods; CA can greatly help improve the accuracy of various phase difference measurement methods and has the most obvious effect on DFT method. However, it can be seen that LRAF had no effect on the DFT-based method but had an effect on the other two methods; CA is helpful for improving the measurement accuracy of various phase difference measurement methods and had the most obvious effect on the DFT-based method.

## 6. Discussion

According to the experimental results in Section 5, both LRAF and CA can effectively filter out noise, but the effect of CA is much better than LRAF. We think that this is mainly because CA makes use of the consistency of the waveform of each period of the sinusoidal signal, but LRAF only uses the method of finding the local average of the adjacent sampling points, and the filtering effect is limited.

Secondly, both LRAF and CA can help the DFT-, DC-, and HT-based phase difference measurement methods improve their measurement accuracy, but they are not very helpful for the DC- and HT-based methods. The main reason may be that the DC- and HT-based phase difference measurement methods themselves have a strong ability to suppress random noise. 

Third, when the SNR is small, both LRAF and CA have obvious filtering effects on the signal, but when the SNR is large, the preprocessing has no effect on the measurement accuracy. That is because LRAF and CA only play the role of filtering or suppressing noise; the noise in the signal is relatively small when the SNR is relatively large, so there is no noise that can be filtered even with LRAF and CA.

Fourth, the number of CA cycles has a great influence on the phase difference measurement results. The higher the number of cycles, the more obvious the filtering effect and the higher the accuracy of the corresponding phase difference measurement. This is in line with the law: the larger the number of samples, the more accurate the measurement results.

In addition, it is worth mentioning that the effects of LRAF and CA were only verified on the DFT-, DC-, and HT-based phase difference measurement methods in this paper, so further work can be done in the future to verify them on other phase difference measurement methods, such as the least squares (LS) method, independent component analysis (ICA) method, and zero cross-detection (ZCD) method.

## 7. Conclusions

In order to solve the phase difference measurement problem of the high-frequency internal calibration signal of the InSAR system, a phase difference measurement method based on LRAF and CA under under-sampling conditions was proposed in this paper, and the sampling frequency selection criteria under the under-sampling condition were determined. Experimental results confirmed the validity of the method. Through theoretical analysis and experiments, the conclusions obtained in this paper are as follows:

(1) The sampling frequency used to under-sample high-frequency sinusoidal signals should meet the conditions in Equations (6) or (11).
(2) Both LRAF and CA can effectively filter out noise, but the effect of CA is much better than LRAF.
(3) Both LRAF and CA can help the DFT-, DC-, and HT-based phase difference measurement methods improve their measurement accuracy, but they are not very helpful for the DC- and HT-based methods.
(4) When the SNR is small (<12 dB under the simulation condition of this paper), both LRAF and CA have obvious filtering effects on the signal, but when the SNR is large, the preprocessing has no effect on the measurement accuracy.
(5) The number of CA cycles has a great influence on the phase difference measurement results. The higher the number of cycles, the more obvious the filtering effect and the higher the accuracy of the corresponding phase difference measurement.

In summary, the phase difference measurement method proposed in this paper is suitable for the phase difference measurement of the high-frequency internal calibration signal of the InSAR system for phase error calibration. This method can effectively filter out noise in the sinusoidal signal, improve the phase difference measurement accuracy of the sinusoidal signal, and greatly reduce the phase error. The simulation experiments in Section 5 demonstrate the effectiveness of the proposed method.

## Figures and Tables

**Table 1:** Parameters used in the experiments.

Parameters	Value Size
signal-to-noise ratio (SNR)	2 dB
signal frequency (f)	200 MHz
sampling frequency ($f_{s}$)	33 MHz
total length (N)	10,240
number of points in one baseband signal period ($N_{0}$)	1024
amplitude of signal 1 ($A_{1}$)	0.25
amplitude of signal 2 ($A_{2}$)	0.2
initial phase of signal 1 ($\varphi_{1}$)	30°
initial phase of signal 2 ($\varphi_{2}$)	45°

**Table 2:** The mean and standard deviation of the measurement error by different phase difference measurement methods.

Measurement Methods	Measurement Error	Original Signal	LRAF Only	CA Only	LRAF and CA
DC-based method	Mean (°)	−0.0281	−0.0276	−0.0235	−0.0217
	Standard deviation (°)	0.6852	0.6391	0.6348	0.6292
DFT-based method	Mean (°)	−0.0644	−0.0601	−0.0365	−0.0305
	Standard deviation (°)	1.9611	1.9578	0.6257	0.6252
HT-based method	Mean (°)	−0.0451	−0.0426	−0.0357	−0.0361
	Standard deviation (°)	0.7114	0.6447	0.6374	0.6278

## References

[1] Bertotti F.L., Hara M.S., Abatti P.J. (2010). A simple method to measure phase difference between sinusoidal signals. Rev. Sci. Instrum..

[2] So H.C., Zhou Z. (2013). Two accurate phase-difference estimators for dual-channel sine-wave model. EURASIP J. Adv. Signal Process..

[3] Vucijak N.M., Saranovac L.V. (2010). A Simple Algorithm for the Estimation of Phase Difference Between Two Sinusoidal Voltages. IEEE Trans. Instrum. Meas..

[4] Yang J.R. (2014). Measurement of Amplitude and Phase Differences Between Two RF Signals by Using Signal Power Detection. IEEE Microw. Wirel. Compon. Lett..

[5] Ignatjev V., Stankevich D. (2017). A Fast Estimation Method for the Phase Difference Between Two Quasi-harmonic Signals for Real-Time Systems. Circuits Syst. Signal Process..

[6] Bai L., Su X., Zhou W., Ou X. (2015). On precise phase difference measurement approach using border stability of detection resolution. Rev. Sci. Instrum..

[7] Chen N., Fan S.C., Zheng D.Z. (2019). A phase difference measurement method based on strong tracking filter for Coriolis mass flowmeter. Rev. Sci. Instrum..

[8] Zhang M., Wang H., Qin H.B., Zhao W., Liu Y. (2018). Phase Difference Measurement Method Based on Progressive Phase Shift. Electronics.

[9] Choi U.G., Kim H.Y., Han S.T., Yang J.R. (2019). Measurement Method of Amplitude Ratios and Phase Differences Based on Power Detection Among Multiple Ports. IEEE Trans. Instrum. Meas..

[10] Werner M., Häusler M. X-SAR/SRTM instrument phase error calibration. Proceedings of the IEEE 2001 International Geoscience and Remote Sensing Symposium.

[11] McWatters D.A., Lutes G., Caro E.R., Tu M. (2001). Optical calibration phase locked loop for the Shuttle Radar Topography Mission. IEEE Trans. Instrum. Meas..

[12] Farr T.G., Rosen P.A., Caro E., Crippen R., Duren R., Hensley S., Kobrick M., Paller M., Rodriguez E., Roth L. (2007). The Shuttle Radar Topography Mission. Rev. Geophys..

[13] Wang Y., Liang X., Wu Y. A comparison of internal calibration schemes for spaceborne single-pass InSAR applications. Proceedings of the IEEE International Geoscience and Remote Sensing Symposium.

[14] Ree J.D.L., Centeno V., Thorp J.S., Phadke A.G. (2010). Synchronized Phasor Measurement Applications in Power Systems. IEEE Trans. Smart Grid.

[15] Wang Z., Mao L., Liu R. (2012). High-Accuracy Amplitude and Phase Measurements for Low-Level RF Systems. IEEE Trans. Instrum. Meas..

[16] Yoon H., Park K. Development of a laser range finder using the phase difference method. Proceedings of the Optomechatronic Sensors & Instrumentation, International Society for Optics and Photonics.

[17] David S.M., Francisco M.M., Ernesto M.G., José L.G. (2016). SNR Degradation in Undersampled Phase Measurement Systems. Sensors.

[18] Shen T., Tu Y., Li M., Zhang H. (2015). A new phase difference measurement algorithm for extreme frequency signals based on discrete time Fourier transform with negative frequency contribution. Rev. Sci. Instrum..

[19] Wang K., Tu Y., Shen Y., Xiao W., Des M. (2018). A modulation based phase difference estimator for real sinusoids to compensate for incoherent sampling. Rev. Sci. Instrum..

[20] Liang Y.R., Duan H.Z., Yeh H.C., Luo J. (2012). Fundamental limits on the digital phase measurement method based on cross-correlation analysis. Rev. Sci. Instrum..

[21] Shen Y.L., Tu Y.Q., Chen L.J., Shen T.A. (2015). Phase difference estimation method based on data extension and Hilbert transform. Meas. Sci. Technol..

[22] Micheletti R. (1991). Phase angle measurement between two sinusoidal signals. IEEE Trans. Instrum. Meas..

[23] Gong G., Lu H., Chen G., Jin M., Chen X. Phase Difference Measurement Method for Sine Signals Based on Fast ICA. Proceedings of the 2013 Fourth Global Congress on Intelligent Systems.

[24] Lin D.Y., Lu J.F., Jia R.C., Yang L. Research on the Technology of Phase Difference Measurement Based on FPGA. Proceedings of the 2014 Fourth International Conference on Instrumentation and Measurement, Computer, Communication and Control.

[25] Oppenheim A.V., Schafer R.W. (2009). Discrete-Time Signal Processing.

